# A commitment renewed

**DOI:** 10.4103/0253-7613.62393

**Published:** 2010-02

**Authors:** R.K. Dikshit

**Affiliations:** Chief Editor, Indian Journal of Pharmacology, Department of Pharmacology, B.J. Medical College, Ahmedabad - 380 016, India E-mail: ijp@ijp_online.com

With this issue, a new editorial team takes over the reins of Indian Journal of Pharmacology. Although the names (and designations) may have changed (a little), the dedication remains the same. The new Chief Editor and the editorial team feel honored in doing a job that has been done so admirably in the past by a number of stalwarts. Today, IJP in its current form is the result of their cumulated wisdom and devotion. We would consider ourselves successful even if we are able to maintain the existing standards, if not improve them. I strongly believe this will be the best tribute to all our worthy predecessors!

We are aware that we have to work on several fronts, from the quantity, quality, and variety of the papers to the new columns and nonreceipt of the journal! I am sure that all these issues can be handled successfully if we continue to get your active cooperation. With the recent inclusion in PubMed (many thanks to our publishers M/s Medknow Publications and Media Pvt Ltd. and especially Dr. D. K. Sahu), the IJP has achieved all that a journal may aspire. It is the right time, friends, to try us first for the publication of your quality works!

None of us is a qualified/professional editor. We have, however, trained ourselves by experience. It is still quite possible that we may be found lacking in several areas. Please bear with us for our omissions. Trust me they are not deliberate! A scientific journal can have only one objective, i.e., to serve the discipline it belongs to. Reviewers, authors, and readers are the backbone of the journal. A journal can only be as good as we are. With this issue, we rededicate ourselves to the cause of pharmacology (and pharmacologists) in India and abroad! Come, friends, join us on a journey towards this noble task.

